# Reverse Sural Flap for Reconstruction of a Distal Leg Soft-Tissue Defect Following Dog Bite Injury: A Case Report

**DOI:** 10.7759/cureus.109811

**Published:** 2026-05-28

**Authors:** Daniel A Portillo-Rodríguez, Brayan J Benguechea-Santiago, Jesus A Vazquez-Calderon, Ivan A Ornelas-Ayala, Dina E Rodríguez-Soto, Cynthia N Cuevas-Lujan, Abril M Peralta-Enriquez

**Affiliations:** 1 Surgery, Instituto Mexicano del Seguro Social, Juárez, MEX; 2 Surgery, Instituto Mexicano del Seguro Social, Juarez, MEX; 3 Medical Education and Health Research, Instituto Mexicano del Seguro Social, Juárez, MEX; 4 Plastic and Reconstructive Surgery, Instituto Mexicano del Seguro Social, Juárez, MEX

**Keywords:** dog bite injury, flap survival, lower limb reconstruction, negative pressure wound therapy, soft tissue defect, sural flap, traumatic infection

## Abstract

Dog bites are a frequent cause of extremity trauma and pose a substantial risk of infection and complications, particularly when bone exposure is present. We present the case of a 58-year-old male with no comorbidities who arrived at the emergency department after being attacked by a dog while cycling, sustaining multiple bites to the left lower extremity and right forearm. On physical examination, the patient exhibited complex wounds measuring up to 10 × 5 cm, with muscular and tibial bone exposure in the left leg but no associated fracture, and additional forearm lesions with muscle exposure but preserved tendon and neurovascular structures. Hemodynamic parameters were stable, while laboratory tests revealed marked leukocytosis (30,000/µL). The patient underwent urgent surgical irrigation, radical debridement, partial wound closure, and empiric intravenous antibiotic therapy on the day of admission. Due to persistent tibial bone exposure, definitive reconstruction with a distally based reverse sural flap was performed five days later, following serial wound care and infection control. The postoperative course was uneventful, with complete flap viability and 95% graft take by postoperative day seven, enabling discharge with oral antibiotics and local wound care. This case underscores the critical importance of prompt surgical and multidisciplinary management in complex dog bite injuries and further supports the reverse sural flap as a reliable, reproducible, and effective reconstructive option for coverage of distal leg defects with bone exposure.

## Introduction

Dog bites account for approximately 85-90% of all animal bites in humans and represent a frequent cause of emergency department visits and extremity trauma. These injuries carry a substantial risk of infectious complications because of polymicrobial contamination and may result in complex soft-tissue defects requiring staged surgical management, including aggressive debridement, antimicrobial therapy, tetanus prophylaxis, and rabies risk assessment when indicated. Common pathogens include *Pasteurella multocida*, *Capnocytophaga canimorsus*, *Staphylococcus aureus*, and anaerobic bacteria. Accurate identification of the biting animal is particularly important for rabies risk assessment, vaccination status evaluation, and public health management. Empiric antimicrobial therapy in dog bite injuries is generally guided by the expected polymicrobial flora commonly associated with canine oral contamination [1].

The reverse sural flap (RSF) is a reliable regional reconstructive option for distal lower-extremity soft-tissue defects with exposed bone or tendon. It provides durable vascularized coverage without the need for microsurgical anastomosis, making it particularly useful in traumatic wounds and resource-limited settings [2].

Reconstruction of distal leg defects remains particularly challenging because of the limited local soft-tissue availability, relatively poor tissue mobility, and the need for durable coverage over exposed bone or tendon. The reverse sural flap is based on retrograde blood flow through distal septocutaneous perforators arising from the peroneal artery, allowing rotation of well-vascularized tissue to the distal third of the leg, ankle, and foot without microsurgical anastomosis. These characteristics make the technique especially valuable for traumatic wounds that require reliable soft-tissue coverage in resource-limited settings.

Published series have reported flap survival rates of approximately 95%, with only a minority of patients requiring reoperation because of superficial necrosis. Favorable outcomes are closely associated with meticulous patient selection, particularly avoiding individuals with significant comorbidities that impair wound healing. In addition, successful reconstruction depends on experienced surgical teams, rigorous preoperative planning, and thorough debridement prior to flap coverage [3].

This case illustrates how, in a resource-constrained clinical setting, a complex dog bite injury with tibial bone exposure was successfully managed using a reverse sural flap, thereby avoiding more complex microsurgical procedures while preserving limb function.

## Case presentation

**Figure 1:**
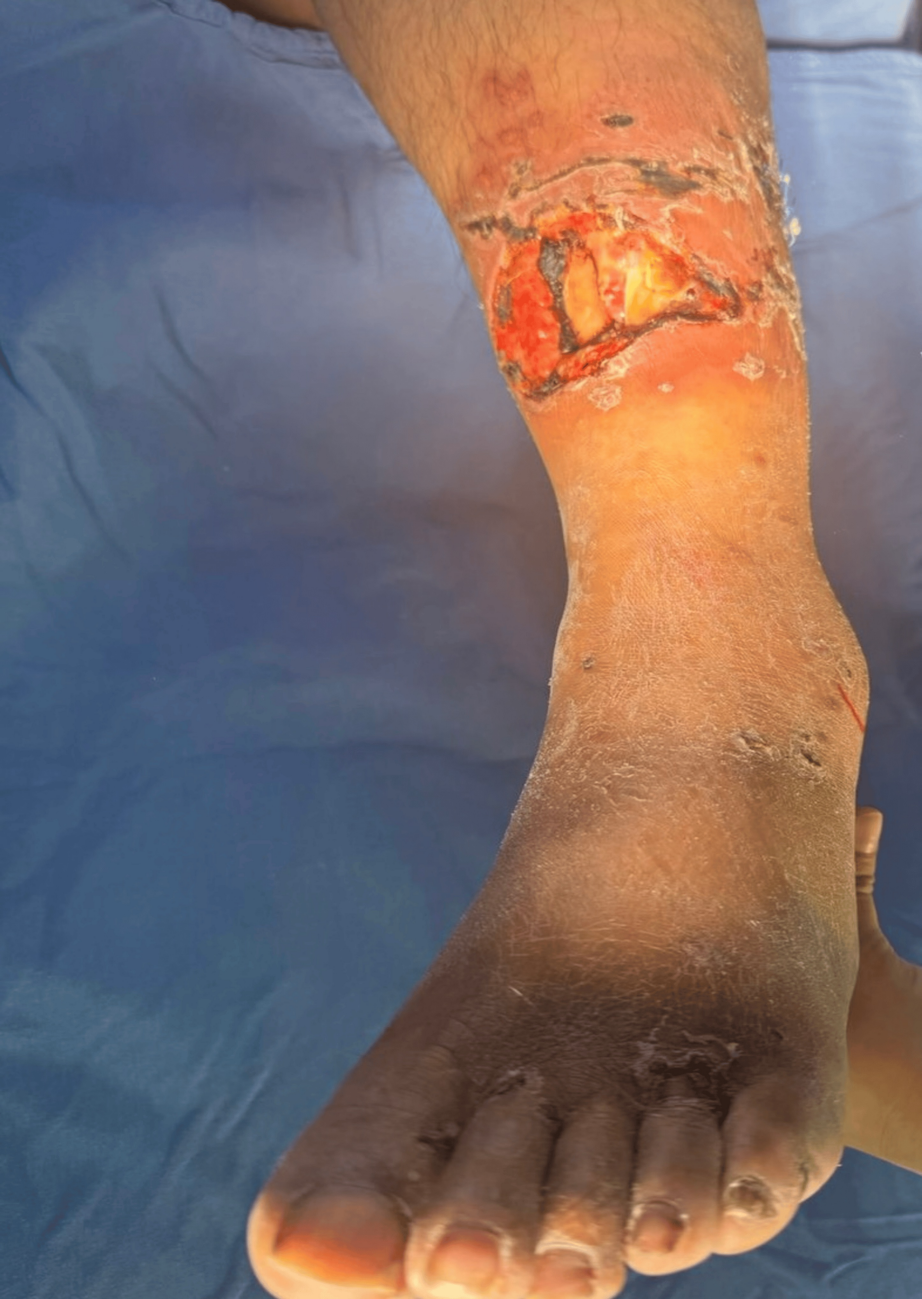
Soft tissue defect in the distal third of the left leg secondary to a dog bite. The wound shows an irregular raw area with loss of skin coverage and anterior tibial bone exposure.

A 58-year-old male patient with no significant past medical history presented to the emergency department after being attacked by a dog while cycling. He sustained multiple bites to the left lower limb and right forearm. On initial examination, complex soft-tissue wounds measuring up to 10 × 5 cm were identified in the left leg with anterior tibial bone exposure (Figure 1), as well as right forearm lesions with muscle exposure but preserved tendon and neurovascular integrity. Vital signs were stable, and the patient was afebrile on admission. Physical examination revealed extensive soft-tissue injury with local edema, erythema, and contamination of the wounds, but without purulent drainage or signs of necrotizing soft-tissue infection. Laboratory tests demonstrated marked leukocytosis (30,000/µL), consistent with a severe inflammatory response secondary to contaminated traumatic injury. Blood cultures were not obtained because the patient remained hemodynamically stable without systemic signs of sepsis. Serial laboratory evaluation showed progressive improvement in leukocytosis following surgical debridement, intravenous antibiotic therapy, and wound management. Tetanus prophylaxis was administered according to institutional protocol. Rabies risk assessment was also performed at presentation. Because the attacking dog was identified, available for veterinary observation, and showed no clinical signs suggestive of rabies infection, neither rabies vaccine prophylaxis nor rabies immunoglobulin administration was indicated according to public health recommendations.

Initial management consisted of urgent surgical irrigation, radical debridement, copious saline lavage, and partial wound closure performed on the day of admission. Empiric intravenous ampicillin-sulbactam therapy was initiated upon admission due to the high risk of polymicrobial contamination associated with dog bite injuries and was maintained throughout the perioperative period for seven days. Following clinical improvement and successful flap coverage, the patient was discharged with oral amoxicillin-clavulanate and local wound care instructions. Despite initial management, persistent anterior tibial bone exposure remained evident. After five days of serial wound care, infection control, and wound-bed optimization, the patient was referred to the plastic and reconstructive surgery team for definitive coverage with a reverse sural flap (RSF).

Surgical technique

**Figure 2:**
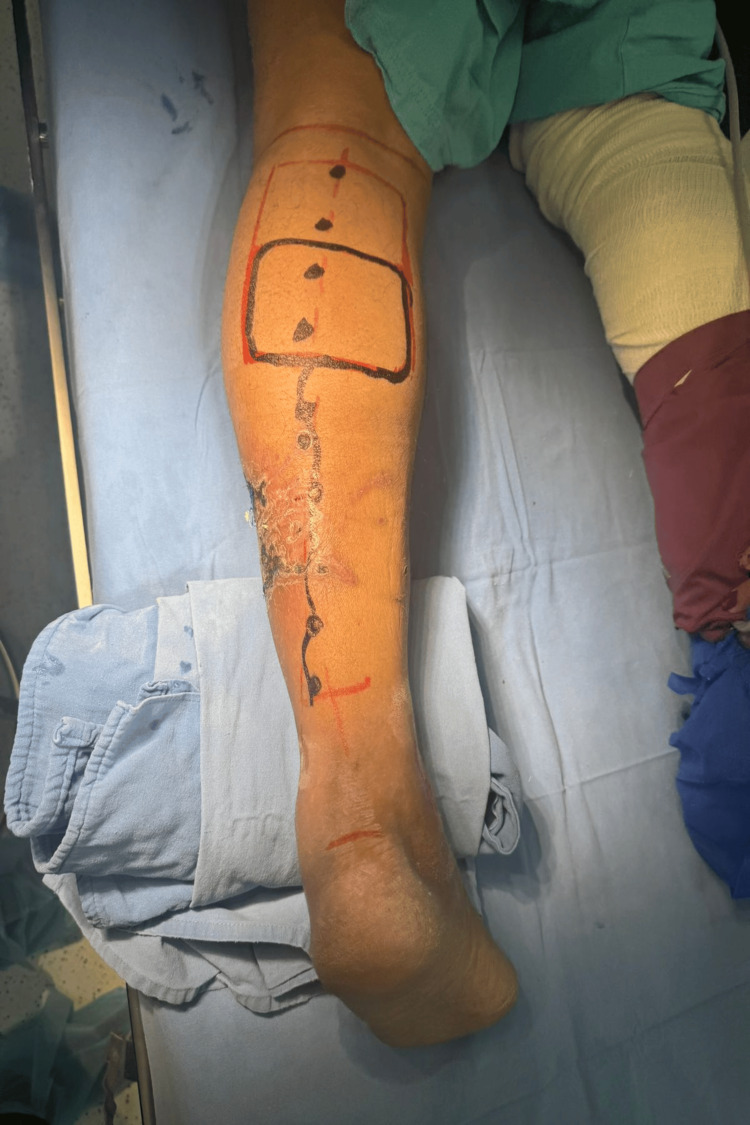
Preoperative planning of the reverse sural flap in the left leg. A rectangular skin paddle is outlined on the posterior calf with surgical ink, along with the sural neurovascular axis extending toward the pivot point proximal to the lateral malleolus.

Preoperative planning included outlining a rectangular skin paddle over the posterior calf, centered along the sural neurovascular axis with a pivot point located 6.5 cm proximal to the lateral malleolus. This pivot point was selected to preserve the distal septocutaneous perforators arising from the peroneal artery, which provide the retrograde vascular supply essential for flap viability and distal rotation (Figure 2).

**Figure 3:**
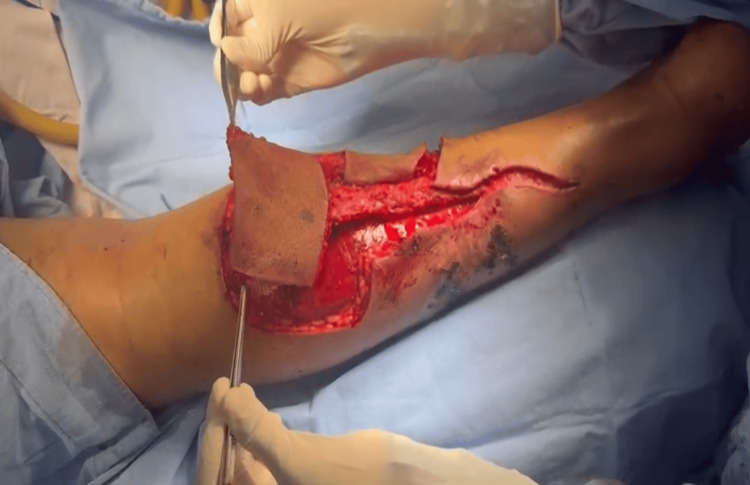
Intraoperative view of the elevation of the reverse sural flap in the left leg. The fasciocutaneous flap is dissected and raised from the posterior calf, including its neurovascular pedicle, and rotated toward the anterior tibial defect with bone exposure.

Under epidural anesthesia and in left lateral decubitus position, a 10 × 8 cm fasciocutaneous skin island was designed and elevated in the subfascial plane from proximal to distal. A wide pedicle measuring approximately 4 cm was preserved to optimize venous drainage and reduce the risk of vascular compromise. The flap was elevated, including the sural nerve, accompanying vascular network, deep fascia, and the lesser saphenous vein, in order to improve venous outflow. Particular care was taken to avoid pedicle kinking, torsion, or compression during flap rotation and inset, thereby minimizing the risk of venous congestion (Figure 3).

**Figure 4:**
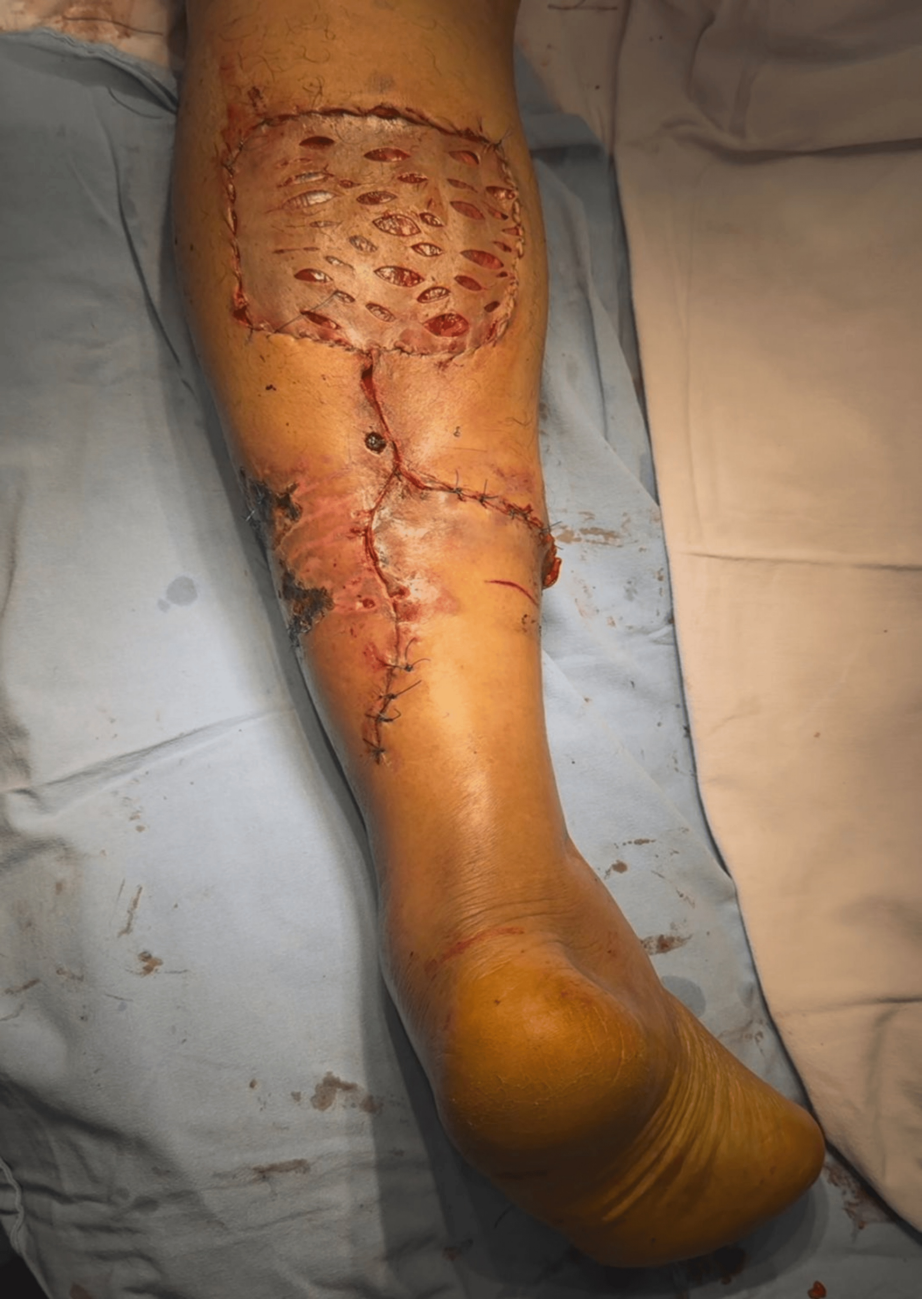
Intraoperative view after rotation of the reverse sural flap to the tibial defect. The proximal donor site is covered with a meshed split-thickness skin graft, and the flap is sutured in two layers to cover the area of bone exposure.

**Figure 5:**
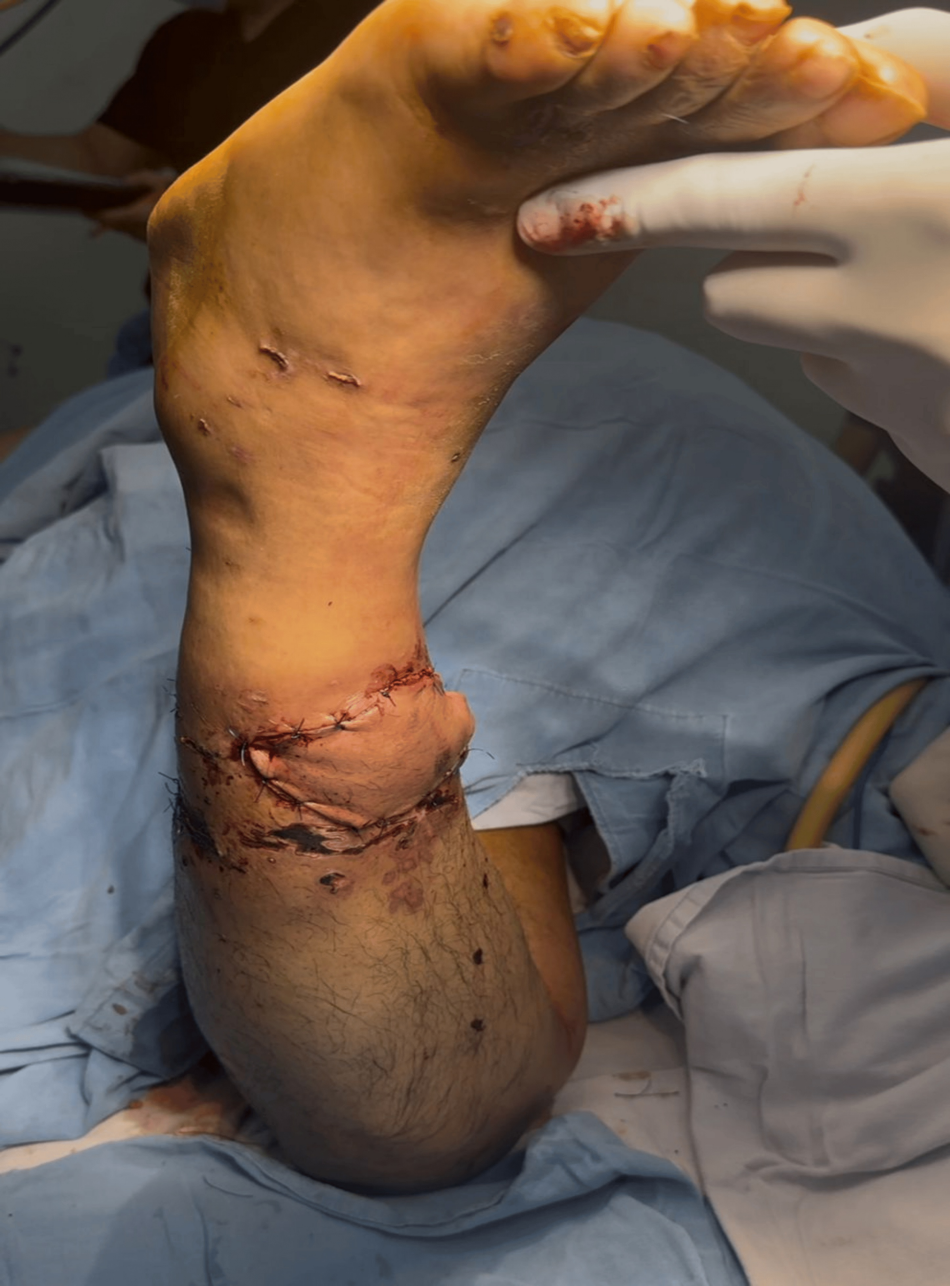
Early postoperative result. The sural flap appears viable with adequate perfusion, and the skin graft is integrating at the donor site without evidence of immediate complications.

The flap was transposed to cover the anterior tibial defect and secured in two layers using absorbable sutures in depth and nylon for the skin (Figure 4). The donor site was reconstructed, with a meshed split-thickness skin graft measuring approximately 0.012 inches in thickness, harvested from the ipsilateral thigh using a dermatome. The graft was secured and covered with paraffin gauze and a compressive bolster dressing to optimize graft adherence and minimize shear forces. Negative-pressure wound therapy (NPWT) was not utilized (Figure 5).

Postoperative course

The postoperative outcome was favorable. The flap remained fully perfused without venous congestion, partial necrosis, or infectious complications. By postoperative day seven, 95% skin graft take and complete flap viability were achieved, allowing hospital discharge with oral amoxicillin-clavulanate and local wound care instructions. At three-month follow-up, the flap remained fully viable with stable soft-tissue coverage and preserved limb contour. The patient achieved independent ambulation without functional limitations and reported satisfactory cosmetic and functional outcomes. Mild hypoesthesia over the sural nerve distribution was present, as expected, but no chronic pain or gait impairment was observed. The donor site demonstrated complete graft integration without dehiscence or hypertrophic scarring, and no late venous congestion, flap necrosis, or recurrent infection developed during follow-up.

## Discussion

In this case, despite the severe contamination and high infectious risk associated with the dog bite injury, the reverse sural flap (RSF) demonstrated full integration and favorable evolution. Although our patient did not present with confirmed osteomyelitis, evidence derived from contaminated traumatic lower-extremity defects remains clinically relevant, as these scenarios share important reconstructive principles, including aggressive debridement, infection control, and the need for durable vascularized soft-tissue coverage. In the study by Luo et al. involving patients with traumatic osteomyelitis and lower-extremity soft-tissue defects, reported outcomes included defect coverage up to 20.5 × 13 cm, bone-healing rates approaching 99%, flap survival rates above 90%, and osteomyelitis recurrence rates of approximately 12% during long-term follow-up [4,5]. These findings support the use of the reverse sural flap after adequate debridement and infection control in selected contaminated traumatic wounds requiring durable vascularized soft-tissue coverage.

Other reconstructive alternatives were also considered based on the defect's location and characteristics. Local perforator or propeller flaps may provide reliable coverage for selected distal leg defects; however, their use can be limited in traumatic wounds with surrounding tissue compromise or uncertain perforator reliability. Soleus or gastrocnemius muscle flaps are valuable options for proximal and middle-third leg defects, but they offer limited reach for distal tibial exposure, such as in our patient. Free tissue transfer remains the gold standard for extensive lower-extremity reconstruction because of its versatility and ability to restore complex composite defects; nevertheless, it requires microsurgical expertise, longer operative time, recipient vessel availability, and specialized postoperative monitoring. Dermal substitutes followed by skin grafting may represent another reconstructive strategy, although they generally provide less durable coverage over exposed bone in high-risk traumatic wounds. In this context, the reverse sural flap represented a practical and reliable reconstructive option with adequate arc of rotation, dependable vascularity, and technical reproducibility.

In the present case, the reverse sural flap was selected because it provided reliable coverage for distal tibial exposure without the need for microsurgical resources or prolonged operative time. Alternative reconstructive options such as free flaps, perforator flaps, propeller flaps, or muscle flaps were considered less favorable given the contaminated traumatic setting and the resource-limited environment. Free tissue transfer offers excellent soft-tissue coverage and contour restoration; however, it requires microsurgical expertise, longer operative times, specialized postoperative monitoring, and suitable recipient vessels, which may not always be readily available in emergency reconstructive settings. In contrast, the reverse sural flap represents a technically reproducible regional flap with dependable vascularity and relatively low donor-site morbidity, making it particularly useful for traumatic distal lower-extremity defects with exposed bone.

The reverse sural flap also offers specific advantages in contaminated wounds because it allows robust vascularized tissue coverage following aggressive debridement while avoiding microsurgical anastomosis in potentially inflamed recipient vessels. Nevertheless, the technique has recognized limitations. Advanced age, diabetes mellitus, peripheral vascular disease, venous insufficiency, and smoking have all been associated with higher rates of venous congestion, partial flap necrosis, and delayed wound healing. Donor-site morbidity, including graft-related contour irregularities, hypoesthesia along the sural nerve distribution, and aesthetic concerns, should also be considered when selecting reconstructive strategies. Compared with microsurgical reconstruction, the reverse sural flap may provide less optimal contour and sensibility restoration; however, it remains a highly valuable alternative in settings where rapid, reliable, and technically accessible reconstruction is required.

The RSF has consistently achieved survival rates around 90-95% and low recurrence of infection, underscoring its value in complex leg and foot reconstructions. Critical steps include debridement down to bleeding bone, elimination of necrotic tissue, and securing a well-vascularized bed to reduce bacterial load. Patient-related factors, such as age over 40 years and advanced Cierny-Mader type IV lesions, have been associated with higher recurrence rates, emphasizing the importance of appropriate case selection and multidisciplinary care [5]. Although our patient did not present with established osteomyelitis, the high infectious risk inherent to a contaminated dog bite parallels scenarios reported in post-traumatic osteomyelitis, making the successful outcome particularly relevant.

Adjunctive measures, such as negative-pressure wound therapy (NPWT), have been described as useful strategies to improve the wound environment, reduce edema, and decrease venous congestion in lower-extremity flap reconstruction [6]. Although NPWT was considered as a potential adjunct in our patient, successful flap viability and graft integration were achieved without its use following meticulous debridement, careful flap handling, and appropriate postoperative wound care. This suggests that while NPWT may provide additional benefit in selected cases, satisfactory reconstructive outcomes can still be achieved in resource-limited settings without its routine application.

The reverse sural flap remains a reliable, versatile, and reproducible option for the coverage of traumatic and chronic defects of the distal leg and midfoot. It offers the advantage of avoiding microsurgical techniques and preserving the major arterial trunks. Nonetheless, its success is closely related to venous outflow, as venous congestion is the most common cause of flap compromise. Strategies proposed to optimize venous drainage include preservation or distal ligation of the lesser saphenous vein, venous supercharging to proximal recipient veins, and inclusion of adipofascial tissue with perforators to improve circulation. Flap dimensions also influence outcomes, with larger designs increasing the risk of circulatory problems; therefore, careful flap design and preservation of adequate pedicle width are considered important to optimize vascular reliability and reduce the risk of venous congestion [7].

## Conclusions

This case highlights the usefulness of the reverse sural flap as a dependable reconstructive option for distal leg defects with bone exposure following contaminated trauma such as dog bite injuries. Early aggressive debridement, appropriate infection control, and careful flap planning were essential for achieving successful reconstructive and functional outcomes. The reverse sural flap remains a practical and effective alternative for lower extremity reconstruction, particularly in settings where microsurgical resources may be limited.

This case demonstrates the successful use of the reverse sural flap for coverage of a distal leg defect with exposed tibia following a contaminated dog bite injury. Adequate surgical debridement, infection control, and careful flap planning allowed durable soft-tissue coverage and satisfactory functional recovery without major postoperative complications during follow-up. The reverse sural flap may represent a reliable reconstructive option in selected traumatic lower-extremity wounds, particularly in resource-limited settings where microsurgical reconstruction may not be readily available.
